# Multimodal imaging study of pancreatic myeloid sarcoma: a case report and literature review

**DOI:** 10.3389/fonc.2023.1259236

**Published:** 2023-09-26

**Authors:** Xianwen Hu, Wenxin Li, Jinyan Tang, Dandan Li, Pan Wang

**Affiliations:** ^1^ Affiliated Hospital of Zunyi Medical University, Department of Nuclear Medicine, Zunyi, China; ^2^ Zunyi Hospital of Traditional Chinese Medicine, Department of Obstetrics, Zunyi, China

**Keywords:** case report, computed tomography, myeloid sarcoma, pancreas, metastasis

## Abstract

Myeloid sarcoma (MS) is a rare extramedullary parenchymal tumor composed of immature myeloid cells, occurring mainly in the lymph nodes, skin, soft tissue, testicles, bones, peritoneum, and gastrointestinal tract, and rarely in the pancreas. Herein, we report the case of a 68-year-old female patient who visited our hospital for medical assistance due to acute abdominal pain. Abdominal computed tomography (CT) and magnetic resonance imaging showed a mass approximately 8 cm in diameter in the pancreatic tail, which was suspected to be a malignant tumor. To further assess the presence of distant metastases, the patient underwent fluorine-18-fluorodeoxyglucose positron emission tomography (^18^F-FDG PET)/CT, which revealed an increased ^18^F-FDG uptake in the corresponding lesions. Subsequently, the patient underwent surgical treatment, and postoperative pathology and immunohistochemistry revealed that the mass was MS. Moreover, we reviewed the clinical features, imaging findings, and histopathology of pathologically confirmed pancreatic MS in the published literature.

## 1 Introduction

Myeloid sarcoma (MS) is a mass formed by tumorigenic myeloid cells and occurs successively or simultaneously with acute myeloid leukemia, myeloproliferative disease, and myelodysplastic syndrome. MS can be divided into two types, depending on whether it is complicated by a hematological disease: extramedullary infiltration of leukemia (leukemic MS) and isolated MS (1). Isolated MS refers to cases in which patients have no blood history at the beginning of the disease, only an extramedullary mass, and MS does not develop into acute myeloid leukemia within 30 days. Owing to the low incidence and non-specific clinical manifestations of this type, 75%–86% of cases reported in the literature are misdiagnosed at the first diagnosis (2). MS can occur in various organs and tissues throughout the body. It is commonly found in the lymph nodes, skin and soft tissues, testes, bones, peritoneum, and gastrointestinal tract, but rarely occurs in areas such as the liver, gallbladder, parotid gland, mediastinum, retroperitoneum, and uterus, and even more rarely in the pancreas (3). Herein, we report a case of pancreatic MS that was pathologically confirmed after surgical resection and review the published literature with the aim of raising awareness about this rare disease.

## 2 Case presentation

**Figure 1:**
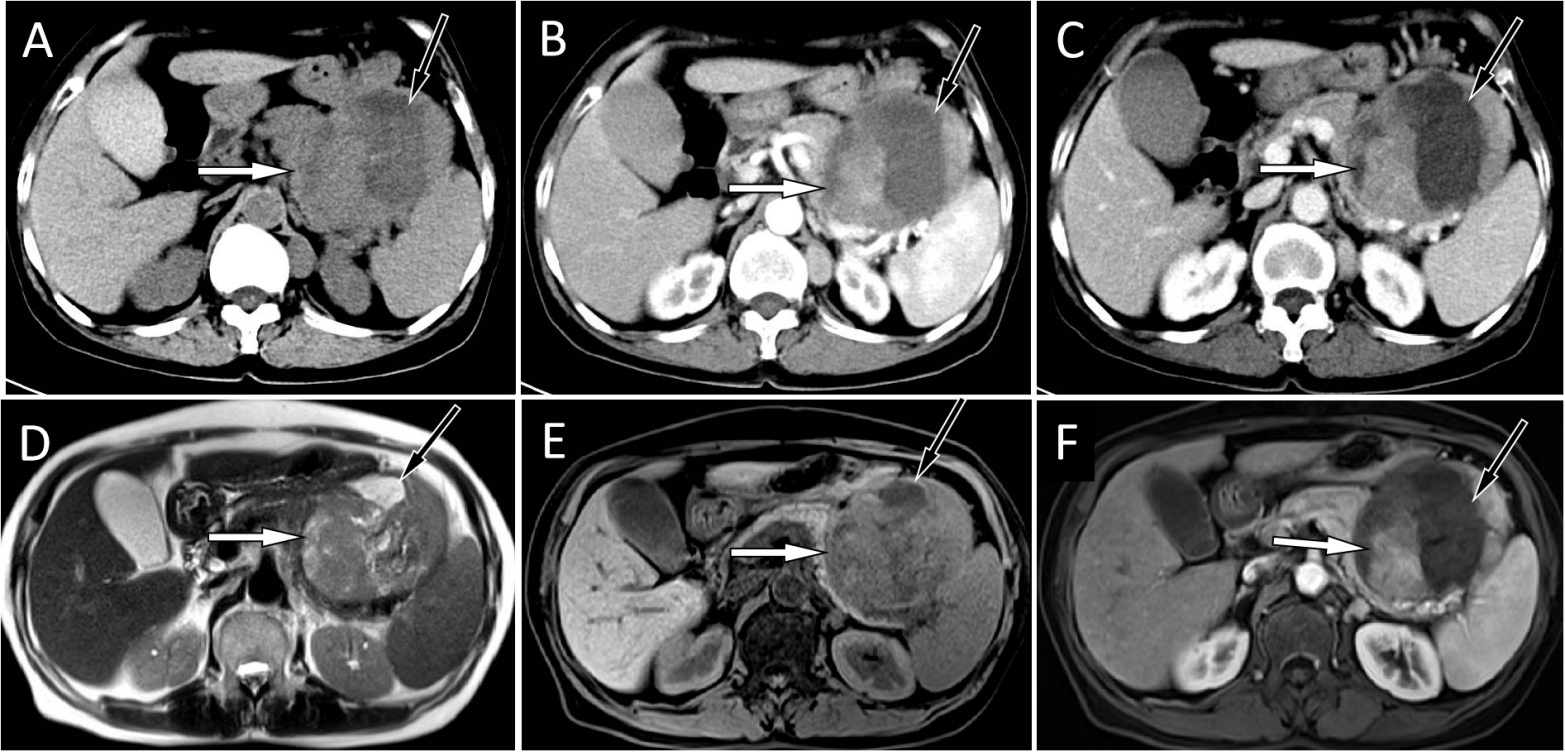
**(A)** An abdominal CT scan showed a large mass located at the tail of the pancreas, with solid components (white arrow) of isodensity and a central cystic area (black arrow). A contrast-enhanced CT scan showed that the solid components (white arrows) of the mass were slightly enhanced at the arterial **(B)** and venous **(C)** stages, and the cystic lesion area was not enhanced (black arrows). The corresponding location showed isosignal on T2WI **(D)** and a slight hyposignal on T1WI **(E)**. On contrast-enhanced T1WI sequences **(F)**, the solid components of the tumor showed mild-to-moderate enhancement (white).

**Figure 2:**
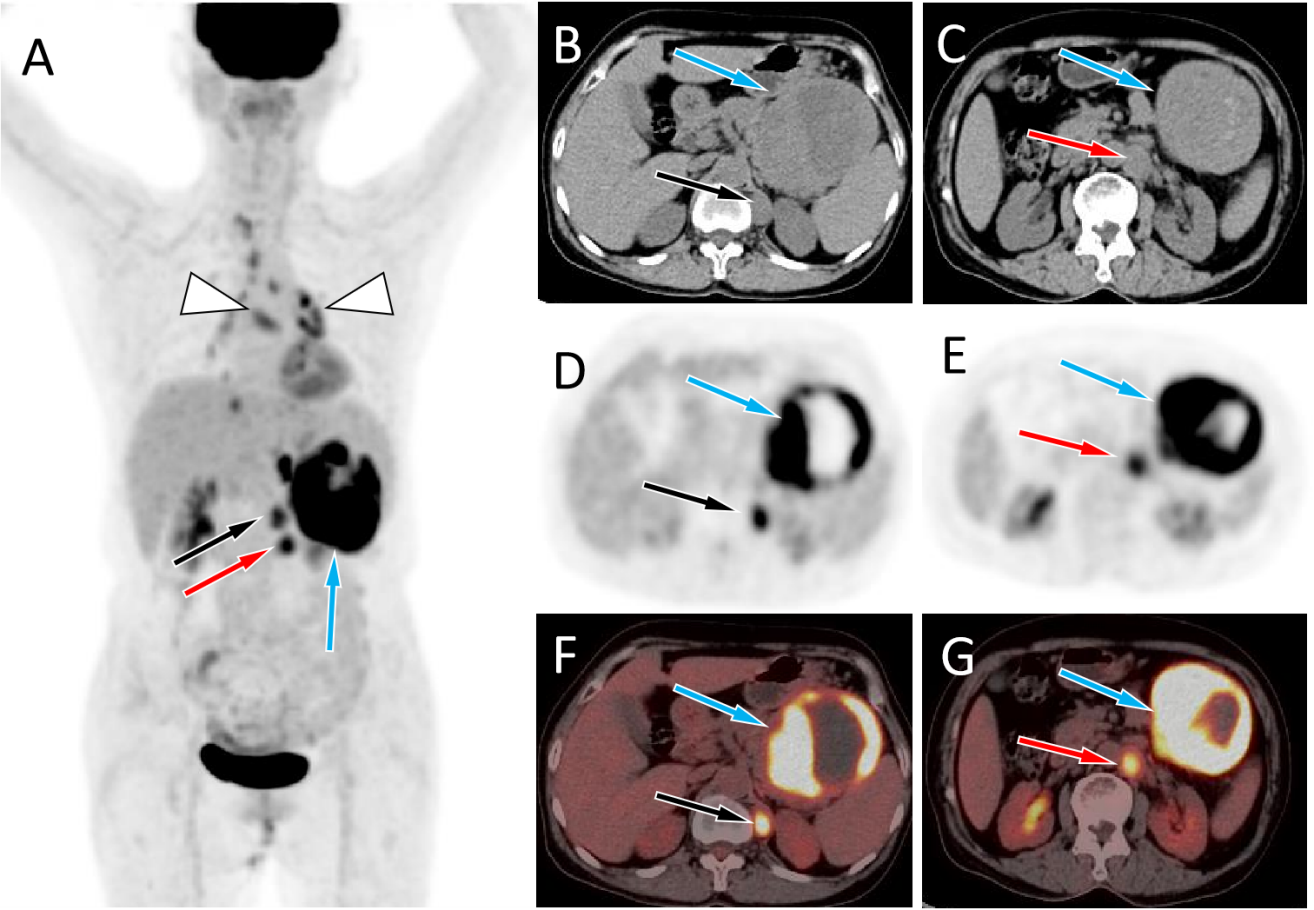
**(A)** The maximum intensity projection of the PET/CT shows a large tumor with increased ^18^F-FDG uptake in the left mid-upper abdomen (blue arrow) and two nodules with increased FDG uptake on its inner side (black and red arrows). Moreover, non-specific inflammatory lymph node uptake of ^18^F-FDG was observed in the mediastinum and hilar area (triangular arrows). Axial images **B, C**, CT; **D, E**, PET; **F, G**, PET/CT fusion images) showed the corresponding large tumor at the tail of the pancreas (blue arrows) and the corresponding two nodules at the left foot of the diaphragm (black and red arrows) and retroperitoneum (red arrows), with increased ^18^F-FDG uptake.

**Figure 3:**
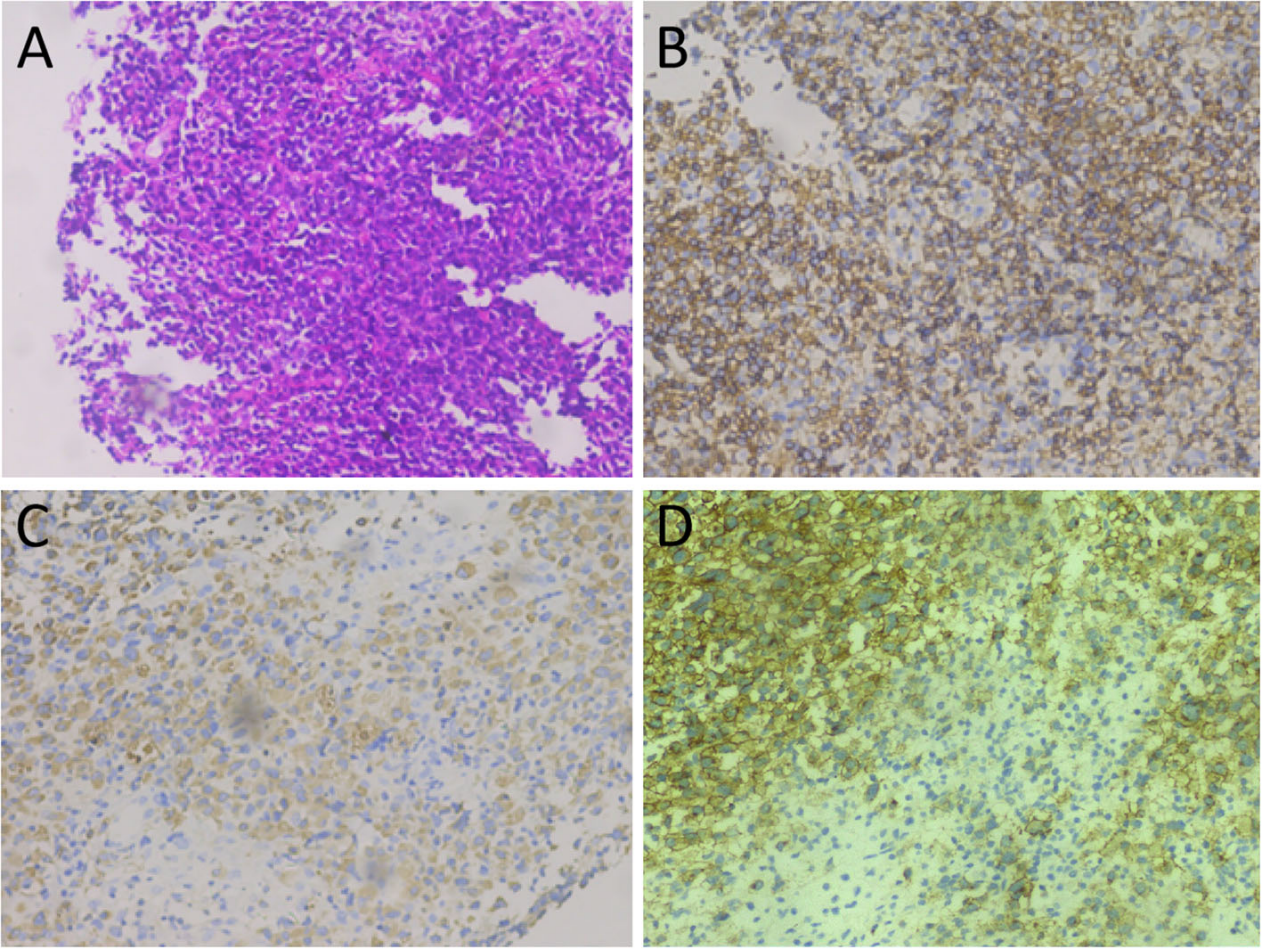
Tumor histopathology and immunohistochemical staining. **(A)** Hematoxylin-eosin staining: microscopically, the tumor cells of relatively uniform size were diffused. **(B–D)** Immunohistochemical staining showed that tumor cells positively expressed MPO **(B)**, CD43 **(C)**, and CD34 **(D)**.

A 67-year-old female patient with no obvious cause of left upper abdominal pain for 3 days was treated at her local county hospital. Laboratory examination showed elevated white blood cells (14.38×10^9^/L, reference range: 3.5–10×10^9^/L), increased hypersensitivity C-reactive protein (237.9 mg/L, reference range: 0–8 mg/L), normal serum amylase levels, and unrelieved left abdominal pain after the local hospital administered anti-inflammatory treatment for suspected acute peritonitis. The patient subsequently visited our hospital for further diagnosis and treatment. Physical examination revealed no other positive signs except tenderness in the patient’s left upper abdomen. Tumor markers of the digestive system, including ferritin, carbohydrate antigen (CA)-724, CA-199, alpha-fetoprotein, and carcinoembryonic antigen, were all within normal reference values. Abdominal computed tomography (CT) and magnetic resonance imaging (MRI) revealed a large cystic solid mass lesion in the tail of the pancreas, without clear boundaries with the surrounding gastric wall and intestines. It was suspected to be a malignant tumor (**Figure 1**). To further assess the presence of distant metastases, the patient underwent fluorine-18-fluorodeoxyglucose positron emission tomography (^18^F-FDG PET)/CT, which showed increased ^18^F-FDG uptake in the peripheral part of the corresponding lesion. Moreover, multiple nodules with increased FDG uptake were observed in the left diaphragm foot and retroperitoneum (**Figure 2**). Based on these imaging findings, the patient was suspected to have pancreatic malignancy with retroperitoneal and left diaphragmatic foot lymph node metastases and underwent tumor tissue resection and lymph node dissection. The resected tumor tissue was sent for pathological examination. Hematoxylin and eosin (H&E) staining revealed small round tumor cells, with eosinophilic particles visible in the cytoplasm, and deeply stained nuclei showing mitotic images (**Figure 3**). Immunohistochemistry (IHC) results showed that the tumor cells positively expressed CD34, CD43, myeloperoxidase (MPO), and P53, and negatively expressed CD117, CD138, CD20, CD3, CD34, CD5, and cytokeratin (CK). Based on these histopathological findings, the patient was diagnosed with pancreatic MS. Owing to the high malignancy of MS, the patient received chemotherapy with cyclophosphamide, doxorubicin, vincristine, and dexamethasone after surgery. Unfortunately, CT examination of the patient 5 months after surgery revealed liver metastases (**Figure S1**), and the patient was subsequently lost to follow-up.

## 3 Literature review

Case reports or case series of pancreatic isolated MS published in the English language until May 1, 2023, were retrieved from the Web of Science, PubMed, and Science Direct databases. After detailed screening of each article, 13 patients in 12 articles met the inclusion criteria (4–15). **Table 1** summarizes the clinical features, imaging findings, and immunohistochemistry of the 14 cases of isolated pancreatic MS (including the present case). Clinical symptoms of isolated pancreatic MS include upper abdominal pain and jaundice. Computed tomography revealed a soft tissue mass with isodensity or slight hypodensity, with or without cystic changes. On MRI, the mass showed a slightly lower signal intensity on T1WI, an equal or slightly higher signal on T2WI, and mild enhancement on contrast-enhanced scans. Similar to our patient, PET/CT of pancreatic MS also showed increased ^18^F-FDG uptake, with a maximum standard uptake value (SUVmax) of 3.5–8.8. Among the cases, IHC tests showed positive expression for MPO (12/12), CD68 (5/5), CD34 (6/8), and CD43 (6/6).

**Table 1:** Clinical features of pancreatic isolated myeloid sarcoma from the literature and our patient.

Case, No.	Author, yr, country	Gender/Age	History	Location	Size (cm)	imaging findings (CT/MRI/PET )	IHC	Treatment	Follow-up (mo)
1(4)	Wu K et al. /2021 /China	M/32	UAP	Head of pancreas	4.4×4.1	Homogeneous mass, mild enhancement*; longer T1 and longer T2 signal; increased FDG uptake.	CD4+, CD10+, CD68+, Bcl-2+, MPO+, CD34+	chemotherapy (IA)	Alive with disease
2(5)	Filizoglu N et al. /20 22/Turkey	M/32	UAP; AML	body and tail of pancreas	NA	Homogeneous mass; increased FDG uptake	MPO+, CD33+, CD117+, and CD34+	NA	NA
3(6)	Tokunaga K et al. /2018/Japan	M/34	UAP	Head of pancreas	10.0×6.0	Hypodense mass with cystic change, mild enhancement*; SUVmax=8.8	CD4+, CD15+, CD33+, CD34+, CD56+, MPO+, CD68+,CD3-, CD7-, CD20-, TdT-, CK(AE1/AE3)-	Chemotherapy(IA)	18/ Alive without disease
4(7)	Breccia M et al. /2003/Italy	F/42	UAP	body and tail of pancreas	NA	Homogeneous mass	CD34+,CD43+, HLA-DR+, CD33+, MPO+, CD45+,CD68+	Chemotherapy(IA)	49/Alive without disease
5(8)	Fukumura Y et al. / 2 021/Japan	M/70	jaundice	Head of pancreas	4.4	Homogeneous mass with cystic change, mild enhancement*	CD33+, MPO+, CD163+, CD68+, CK(AE1/AE3)-, CD34-, CAM5.2-, TdT-, CD20-, CD79a-, CD3-.	None	0.25/died
6(9)	Al-Obaidi A et al. /20 20 /USA	F/57	UAP; jaundice	Head of pancreas	2.2 × 1.5	Homogeneous mass, mild enhancement*	CD43+, CD68+,CD3-, CD20-, CD34-	pancreatectomy and chemotherapy	NA
7(10)	Ishii A et al. /2016/ Japan	M/19	UAP	Head of pancreas	NA	Homogeneous mass; DWI shows limited diffusion; SUVmax=3.5	CD34+, MPO+	Chemotherapy	NA
8(11)	Ravandi-Kashani F et al. /1999/USA	M/31	UAP	Head of pancreas	7	Hypodense mass with cystic change	MPO+, lysozyme+, CD20-, CD3-, CD56-, and TdT-	Chemotherapy (IA+ATRA+G-CSF)	Alive with disease
9(11)		F/61	UAP	Head of pancreas	5	Hypodense mass	CD11a+, CD13+, CD18+, CD45+, CD54+, CD45+, CyA-	Chemotherapy (IA+lisofylline)	Died
10(12)	Rong Y et al. /2010/China	M/40	jaundice, weight loss	Head of pancreas	NA	Hypodense mass	CD43+, MPO+, CD20-, CD2-	pancreatectomy and chemotherapy	Alive without disease
11(13)	Servin-Abad L et al. /2003/USA	M/64	Hematemesis, chest discomfort	Head of pancreas	4.0×4.0	Isodense mass with cystic change	MPO+, CD20-	Chemotherapy(mitoxantrone and etoposide)	12/Died
12(14)	Zhu T/2018 /China	M/36	UAP	tail of pancreas	NA	Hypodense mass; increased FDG uptake	P53+, CD43+, MPO+, CK(AE1/AE3)-, CD56-, CgA-, Syn-, Bcl-2-, Bcl-6-, CD10-, CD20-, CD3-, MUM1-	pancreatectomy and chemotherapy	30/Alive with disease
13(15)	Li XP/2011 /China	F/48	UAP, anemia	Tail of pancreas	4.5×4.0	Hypodense mass with cystic change, mild enhancement*	MPO+, CD20-	pancreatectomy	3/died
15	our case	F/67	UAP	Tail of pancreas	8.0×6.2	Slightly hypodense mass with cystic change; isosignal on T2WI and slightly hyposignal on T1WI; increased FDG uptake, SUVmax=10.4	MPO+,CD34+ CD45+, CD43+, CD117- CD138- CD20- CD3- CD5- CK-	pancreatectomy	5/Alive with disease

AML, acute myeloid leukemia; MPO, myeloperoxidase; TdT, terminal deoxynucleotidyl transferase; CyA, cytokeratin; IA, idarubicin +cytarabine; UAP, upper abdominal pain; * This means that the contrast enhanced scan of the mass is less enhanced than surrounding normal pancreatic tissue; DWI, Diffusion-weighted imaging; ATRA, all-trans-retinoic acid; G-CSF, granulocyte colony-stimulating factor; CT, computed tomography; F, female; M, male; NA, not applicable; Syn, synaptophysin.

## 4 Discussion

MS is also known as green tumor, granulocytic sarcoma, or myeloid cell sarcoma (3). According to the literature, adult MS mainly occurs in the skin (28.2%), lymph nodes (16.3%), testes (6.5%), intestines (6.5%), bones (3.25%), and central nervous system (3.25%) (3). MS rarely occurs in the parotid gland, mediastinum, liver, gallbladder, retroperitoneum, and uterus, and is even rarer in the pancreas (1). To the best of our knowledge, only 13 cases of isolated pancreatic MS have been reported to date. Including this case, a total of 14 cases of isolated pancreatic MS have been reported; 9 were in males and 5 in females, with onset ages ranging from 19 to 70 years. The clinical manifestations of pancreatic MS are mainly related to the location of the mass. Tumors located in the tail of the pancreas cause upper abdominal pain, whereas those located in the head of the pancreas can also present with jaundice and hematemesis. As the disease progresses, patients may exhibit signs of cachexia, such as anemia and weight loss. The patient in the present case was an older adult woman, with a mass located in the tail of the pancreas, whose clinical symptom was upper abdominal pain, which is similar to the clinical symptoms of other pancreatic malignancies without specificity.

Imaging examinations, including CT, MRI, and ^18^F-FDG PET/CT, are essential and helpful for determining the correct preoperative diagnosis and establishing treatment plans. However, there are currently very few reports on the imaging findings of pancreatic MS. Pancreatic MS appears as a homogeneous slightly hypodense or isodense mass on non-contrast-enhanced CT and is prone to cystic change when the mass volume is large (12, 14, 15). On contrast-enhanced CT, it appears as a mild enhancement (4, 8, 9). There are only a few literature reports on the MRI signs of pancreatic MS, which include a slightly lower signal on T1WI, equal or slightly higher signal on T2WI, and limited diffusion on DWI (4, 10). On contrast-enhanced T1WI, MS showed uniform enhancement, with a degree of enhancement similar to that of skeletal muscle (16). MS tumor tissues have a high degree of malignancy and high levels of metabolism; therefore, PET/CT can localize the tumors with an increased ^18^F-FDG uptake (4, 6, 10, 14). Our patient showed a large slightly hypodense soft tissue mass with cystic changes on CT, and slightly longer T1 and T2 signals on MRI with mild enhancement. On PET/CT, the mass showed a high concentration of ^18^F-FDG, with an SUVmax of 10.4, which is consistent with previous reports. Pancreatic MS needs to be differentiated from other tumors, including pancreatic cancer, serous cystadenoma, and solid pseudopapillary tumors, according to its clinical and imaging characteristics. Pancreatic cancer usually presents as a slightly enhanced, equal-, or slightly low-density mass. However, CA199, a serum tumor marker, is usually positive in MS. Pancreatic serous cystadenoma often presents as a multilocular cystic mass, and the cystic lesions are divided into several cystic cavities by thin fibrous septa. Fibrous septa can aggregate into central scars with calcifications, which are characteristic manifestations of serous cystadenoma (17). Solid pseudopapillary tumors of the pancreas are more common in young women, are usually located in the body and tail of the pancreas, and exhibit progressive enhancement on contrast-enhanced CT or MRI as a relatively specific characteristic (18).

However, owing to the rarity and non-specific imaging features of isolated pancreatic MS, accurate diagnosis thereof still relies on histopathological examination. The pathological feature of MS is that the tumor generally appears light green with clear boundaries but without a capsule. Microscopically, immature tumor cells of a relatively consistent size are widely distributed with infiltrative growth, with some tumor cells arranged in a single row of soldier-like bamboo nodes (3). There is almost no consensus on the immunohistochemical diagnosis of MS. MPO, lysozyme, CD68, and other myeloid cell-related markers are the most sensitive and effective markers of MS and can confirm the diagnosis in more than 90% of MS cases (12, 19). In addition, tumors may be positive for CD43, CD117, and CD45 expression, but their specificity is not strong, whereas T and B lymphocyte-related markers, such as CD3, CD20, and CD79a, are negative (19). Based on the current patient and previously published data, immunohistochemistry of pancreatic MS positively expressed MPO (12/12), CD68 (5/5), CD34 (6/8), and CD43 (6/6) at rates of 100%, 100%, 75%, and 100%, respectively.

Treatments for MS include surgical resection, chemotherapy, radiotherapy, and bone marrow transplantation (20). Although a reasonable treatment plan for isolated MS of the pancreas is not yet clear, previous studies have reported that early chemotherapy is a more reasonable treatment option for myelosarcoma and is superior to surgery and radiation therapy in delaying disease progression and improving the overall survival rate (11, 20). However, in most isolated pancreatic cases, including the present case, the final diagnosis is made through histopathological examination after surgical resection of the lesion. Endoscopic ultrasound and fine-needle aspiration are effective methods for identifying pancreatic masses before treatment, which can avoid unnecessary invasive surgical interventions and complications, thereby improving prognosis (21). According to previous studies, the overall prognosis of pancreatic MS is poor, with most patients dying or progressing to acute myeloid leukemia within a few months of diagnosis. Only two patients survived disease-free until the end of follow-up after receiving stem cell transplantation treatment (7, 12). Therefore, stem cell transplantation therapy may be a way to improve the prognosis of pancreatic isolated MS, but the data are limited, and further research is needed.

## 5 Conclusion

In summary, the current study presents multimodal imaging features, including CT, MRI, and PET/CT, of a rare tumor, isolated pancreatic MS. A large isodense or slightly hypodense mass on CT, with slightly longer T1 or T2 signals on MRI, and mild enhancement on contrast-enhanced scans, as well as increased ^18^F-FDG uptake on PET/CT, are of value in the diagnosis of pancreatic isolated MS. The prognosis of pancreatic isolated MS is poor, and future research is needed to explore new treatment methods to improve its prognosis.

## Data availability statement

The original contributions presented in the study are included in the article**Supplementary Material**. Further inquiries can be directed to the corresponding authors.

## Ethics statement

Written informed consent was obtained from the individual(s) for the publication of any potentially identifiable images or data included in this article.

## Author contributions

XH: Conceptualization, Writing – original draft, Writing – review & editing. WL: Data curation, Methodology, Writing – original draft. JT: Formal Analysis, Writing – original draft. DL: Project administration, Writing – original draft. PW: Supervision, Writing – review & editing.
