# Supplementary material for: Multimodal imaging study of pancreatic myeloid sarcoma: a case report and literature review
Source: Front Oncol. 2023 Sep 26;13:1259236. doi: 10.3389/fonc.2023.1259236 (PMC10565847; doi:10.3389/fonc.2023.1259236)
Supplement: Supplementary file 1 [file DataSheet_1.docx]

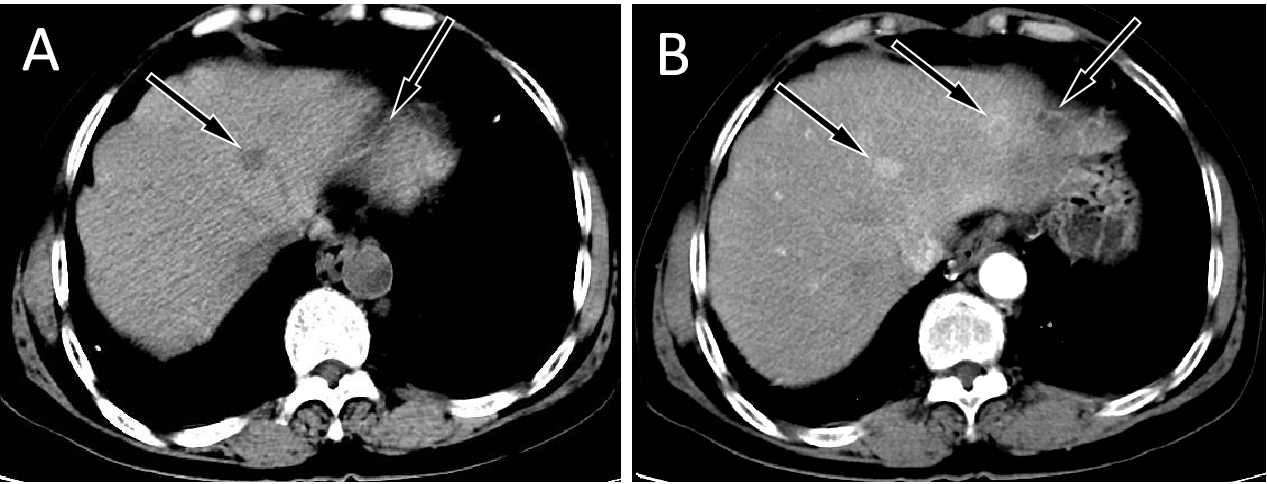


Figure S1. CT examination of the patient 5 months after surgery showed multiple low-density nodules in the left lobe of the liver, and contrast-enhanced CT revealed obvious ring-enhancement of the mass.
